# Japanese Pharmacists’ Perceptions of Self-Development Skills and Continuing Professional Development

**DOI:** 10.3390/pharmacy11020073

**Published:** 2023-04-11

**Authors:** Kayoko Takeda Mamiya, Kiyoshi Takahashi, Tatsuyuki Iwasaki, Tetsumi Irie

**Affiliations:** 1 Department of Pharmaceutical Education, Faculty of Pharmaceutical Sciences, Hokkaido University of Science, Sapporo 0068585, Japan; 2 Department of Pharmaceutical Packaging Technology, Faculty of Life Sciences, Kumamoto University, Kumamoto 862-0973, Japan

**Keywords:** self-development, pharmacists, Japan

## Abstract

Background: The role of healthcare professionals, including pharmacists, is changing. Lifelong learning and continuing professional development (CPD) are more critical than ever for both current and future pharmacists in the face of global health challenges and new technologies, services and therapies that are continually and rapidly introduced into their daily practice. Currently, Japanese pharmacists’ licences are not renewable, although most developed countries have a renewal system. Therefore, understanding Japanese pharmacists’ perceptions of CPD is the first step in reviewing undergraduate and postgraduate education. Methods: The target population was Japanese pharmacists, i.e., community pharmacy pharmacists and hospital pharmacists. The participants were administered a questionnaire with 18 items related to continuing professional development. Results: Our study found that regarding item “Q16 Do you think you need further education in your undergraduate education to continue your professional development?”, (a) the ability to identify one’s own problems and issues, (b) the ability to make plans to solve problems and issues, (c) the ability to carry out plans to solve problems and issues and (d) the ability to repeat steps of self-development, approximately 60% of pharmacists answered that these aspects were “necessary” or “quite necessary”. Conclusion: As part of universities’ responsibility for the lifelong education of pharmacists, it is necessary to systematically conduct teaching seminars or undergraduate education or postgraduate education on self-development while training pharmacists to meet the needs of citizens.

## 1. Introduction

Background: The role of healthcare professionals, including pharmacists, is changing due to changing needs. In particular, the COVID-19 pandemic has forced people worldwide to change their lifestyles and has raised awareness about infection prevention measures among the public, including healthcare professionals [1,2,3,4,5,6,7,8,9]. A previous study investigated infection prevention awareness before and after the spread of COVID-19 among 300 Japanese [1]. In that survey, a total of 89.7% of respondents said that the COVID-19 outbreak had increased their awareness of infection prevention and increased their routine infection control practices following the outbreak. In reality, however, many people cannot answer correctly about infection prevention, with 47.7% of respondents stating that they would like to receive education and training from healthcare professionals on infection prevention methods and their effectiveness [1]. Another survey found that while more Japanese pharmacists feel that the role of pharmacists in the community has changed, only some pharmacists are confident in their ability to instruct patients and the public when asked about infectious diseases and infection prevention methods. Therefore, pharmacists who responded “not confident” also indicated that they would like to engage in ongoing learning due to a lack of knowledge about new infectious diseases and infection prevention methods, highlighting the professionalism of pharmacists [10].

Lifelong learning and continuing professional development (CPD) are more critical than ever critical for both current and future pharmacists in the face of global health challenges as well as new technologies, services and therapies that are continually and rapidly introduced into their daily practice [11,12]. In 2002, the International Pharmaceutical Federation (FIP) defined CPD as “the responsibility of individual pharmacists for systematic maintenance, development and broadening of knowledge, skills and attitudes, to ensure continuing competence as a professional, throughout their careers”. CPD is an ongoing cyclical process involving self-appraisal, the development of a personal learning plan, taking action or implementing the learning plan and evaluation [12]. The FIP published the *Handbook of FIP Global Advanced Development Framework (GADF)* in 2020. The FIP GADF version 1 is a validated tool intended to support the professional development and recognition of the pharmacy workforce everywhere [13]. The framework has the primary purpose of identifying broad areas for professional development and advancement for pharmacists and pharmaceutical scientists to develop their careers in a structured manner [13].

A pilot study of physician graduates of the University of California, San Francisco (UCSF) found that disciplinary action taken against physicians by the Medical Board of California was associated with prior unprofessional behaviour when the physicians were students [14]. In another study, disciplinary action among practising physicians by medical boards was strongly associated with unprofessional behaviour in medical school. Students with the strongest association were those who were described as irresponsible or as having a diminished ability to improve their behaviour [15]. These backgrounds also show the importance of behaviour change associated with self-development or reflection in undergraduate education.

Purpose of this study: Currently, Japanese pharmacists’ licences are not renewable, although most developed countries have a renewal system. In addition, in Japan, to become a pharmacist, students must currently graduate from a pharmacy undergraduate education program (Bachelor of Science) and then take and pass the national pharmacist examination [16]. Thereafter, qualifications such as Specialist Pharmacist or Certified Pharmacist can be obtained at their own will, but are not obligatory. Moreover, various countries provide advanced pharmacist education using education tools such as the GADF [17], but Japanese pharmacists’ education after undergraduate graduation does not involve an integrated education system that considers their recognition or morale as pharmacists. Therefore, it is important for Japanese pharmacists to conduct CPD, self-development independently or organisationally, although this is not sufficient. In this study, we investigated Japanese pharmacists’ recognition of CPD and their recognition of their qualities or competencies in regard to CPD.

Significance of the study: Understanding Japanese pharmacists’ perceptions of CPD is the first step in reviewing undergraduate and postgraduate education. In addition, there is a need for undergraduate education that enables students to undertake CPD after graduation. We believe that even in countries with a licence renewal system, this study can provide useful information for the self-development required in undergraduate education.

## 2. Materials and Methods

Research design: This was a survey study with a target population of Japanese pharmacists, i.e., community pharmacy pharmacists and hospital pharmacists. The number of pharmacists in Japan in 2020 is 321,982 (Supplementary Materials Figure S1). According to 2018 data, of the approximately 310,000 pharmacists, 180,000 are employed as community pharmacists and 54,000 as hospital pharmacists. The remainder is pharmaceutical wholesalers, manufacturers and distributors, universities and others [18,19]. Most pharmacists are therefore employed as either community or hospital pharmacists. The survey company included community pharmacists or hospital pharmacists in the register, and no other pharmacists were included. Then the survey population should include around 400 pharmacy pharmacists and hospital pharmacists each from a statistical point of view. However, the survey company requested that the number of hospital pharmacists and pharmacy pharmacists should be around 200 and 300, respectively, as the number of pharmacists the survey company could handle. The participants were administered a questionnaire with 18 items related to continuing professional development. The respondents were required to understand the content of the questionnaire, and only those who provided consent advanced through the screening process. Recruitment and participants: From January to February 2023, a web-based survey was provided to pharmacist members registered through NEXTIT Inc. in Kobe, Japan [20].

Data analysis: Principal component analysis or general descriptive statistics were used for analysis and were conducted using SPSS Statistics 25.0 (IBM, Chicago, IL, USA).

Ethical considerations: This study was approved by the Institutional Review Board of the Faculty of Pharmacy, Hokkaido University of Science (IRB approval no.: 22–17; date: January 2023). Protection of personal information: As this is an anonymous survey, the data processed in the web-based questionnaire do not contain any personal information and cannot be used to identify individuals. Data files were coded so that only research personnel could access the data on specific computers.

## 3. Results

### 3.1. Characteristics of Respondents

The demographics of the respondents are provided in Table 1 (Q1–12). The total sample consisted of 529 pharmacists, including 321 (60.7%) community pharmacy pharmacists and 208 (39.3%) hospital pharmacists. Regarding the length of time as a pharmacist, the distribution of the respondents was as follows: 7 (1.3%), 1–5 years; 53 (10.0%), 6–10 years; 93 (17.6%), 11–15 years; and 376 (71.1%), more than 16 years. Qualifications such as Specialist Pharmacist or Certified Pharmacist can be obtained at the person’s own will, but are not obligatory. Therefore, we decided to ask about their willingness and current situation as Q4. “Nothing” was selected by 104 respondents (19.7%). We investigated their recognition in Q5–Q7. For “Q6 Do you feel that society’s needs for pharmacists are changing?”, 424 (80.2%) respondents perceived a change, while for “Q5 Are you aware that the model core curriculum for pharmacy education for undergraduate students (6-year pharmacy education) will be revised in 2024?”, 334 (63.1%) pharmacists answered “no”. Regarding recognition of their work as pharmacists, “Q7 Do you feel the need to streamline your work with objects and enhance your work with people?”, 493 (93.2%) respondents answered “yes”. For the questionnaire item “Q9 When you were a student, did you study independently on a daily basis in addition to your assignments?”, 262 (49.5%) respondents answered “I did”, and for “Q10 Do you think continuing self-development is necessary as a pharmacist?”, 518 (97.9%) respondents answered “yes”. Concerning “Q11 How much time do you spend on continuing self-development related to pharmacist work during a week?”, 350 (66.2%) respondents answered 1–3 h; 76 (14.4%) respondents answered 4–6 h; 27 (5.1%) respondents answered 7–9 h; 27 (5.1%) respondents answered more than 10 h and 49 (9.3%) respondents responded 0 h. Regarding “Q12 Are you constantly developing yourself as a pharmacist?”, 133 (25.1%) respondents answered “not at all”, “not much” or “not very much”, and 396 (74.9%) answered “a little”, “somewhat” or “quite a lot”.

### 3.2. Self-Development Initiatives

**Figure 1:**
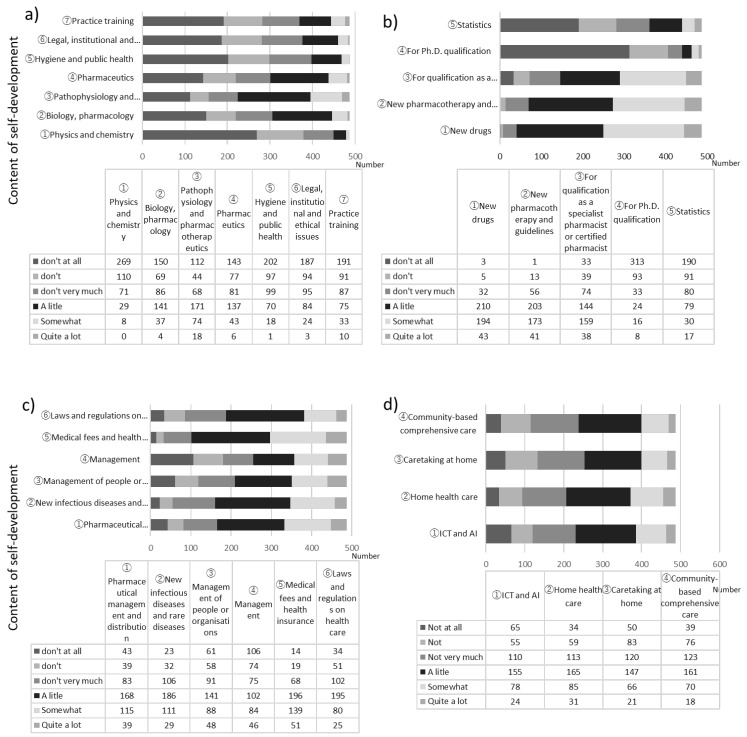
Q13 Content of self-development (*n* = 487). (**a**) Content related to undergraduate education, (**b**) content related to postgraduate expertise and specialisation, (**c**) content related to administration and management and (**d**) content related to adapting to the needs of the times.

We investigated the self-development activities of pharmacists, and the results are shown in Figure 1. Regarding “Q13 In what areas do you engage in self-development? (except for respondents (*n* = 42) of answered “not at all or not much in Q12”)”, we identified four categories: content related to undergraduate education, content related to postgraduate expertise and specialisation, content related to administration and management and content related to adapting to the needs of the times. Regarding the number of self-development activities, content related to undergraduate education was naturally lower than content related to postgraduate expertise and specialisation. In particular, new drugs or new pharmacotherapies and guidelines were undertaken by most pharmacists. Regarding “content related to administration and management” and “content related to adapting to the needs of the times”, more than 50% of respondents had undertaken some form of self-development.

Regarding ICT (Information and Communication Technology) or AI (Artificial Intelligence), 230 (47.2%) respondents answered “not do self-development”, while they feel the need to streamline their work with objects and enhance their work with people. Moreover, with regard to “Q14 How do you prepare yourself for self-development? (for All)”, 230 (43.4%) respondents selected “I search for opportunities on my own to engage in the studying I need to do”. The next most common response was that self-development was implemented as necessary if work problems were identified, which was chosen by 124 (23.4%) respondents. The next was “I voluntarily select information available in the workplace and attend learning sessions outside the workplace”, with 77 (14.6%) respondents.

Regarding Q15, tools for continuing self-development, 184 (34.8%) respondents answered “at home using books purchased by themselves and web searches”, 128 (24.2%) respondents answered “study meeting of medical associations such as Japanese Society of Hospital Pharmacists or Japan Pharmaceutical Association” and 57 (10.8%) respondents answered “pharmaceutical companies (wholesalers) study meeting”.

### 3.3. Developing Self-Development Skills in Undergraduate Education

**Figure 2:**
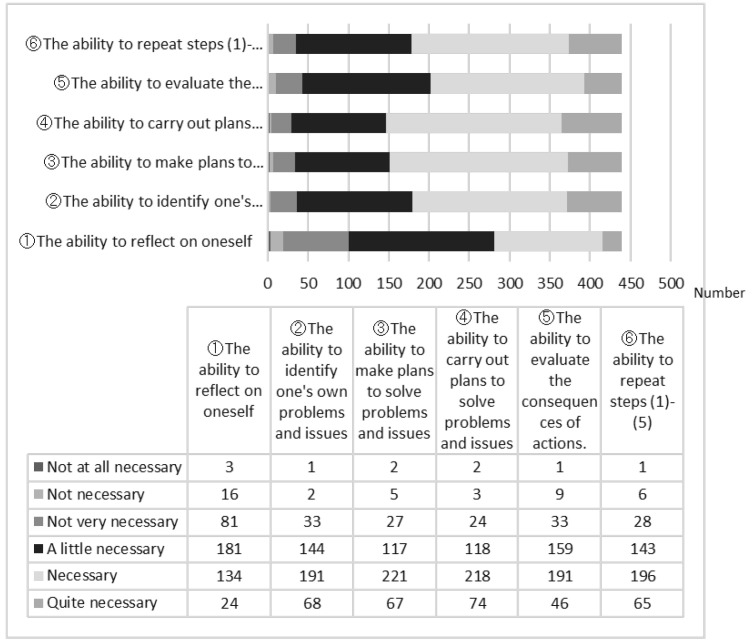
Q17. What other skills do you think were needed in your undergraduate education? (*n* = 439).

Regarding “Q16 Do you think you need further undergraduate education to continue professional development?”, 33 respondents answered “not at all necessary” (6.2%), 57 respondents answered “not necessary” (10.8%), 146 respondents answered “not very necessary” (27.6%), 145 respondents answered “a little necessary” (27.4%), 106 respondents answered “necessary” (20.0%) and 42 respondents answered “quite necessary” (7.9%). With the exception of those who answered “not at all necessary” or “not necessary” (*n* = 90), we asked the other respondents “Q17 What other skills do you think are needed in your undergraduate education?” The results are shown in Figure 2 (*n* = 439). We asked about the following items: (i) the ability to reflect on oneself, (ii) the ability to identify one’s own problems and issues, (iii) the ability to make plans to solve problems and issues, (iv) the ability to carry out plans to solve problems and issues, (v) the ability to evaluate the consequences of actions, and (vi) the ability to repeat steps (i)–(v). For all items, “not at all necessary” was rarely selected for required undergraduate education. In particular, regarding (ii) the ability to identify one’s own problems and issues, (iii) the ability to make plans to solve problems and issues, (iv) the ability to carry out plans to solve problems and issues and (vi) the ability to repeat steps (i)–(v), approximately 60% of pharmacists answered “necessary” or “quite necessary”.

### 3.4. Factors of Continuing Self-Development Skills

To identify Japanese pharmacists’ competencies in continuing self-development, we conducted the principal component analysis using Q5, 6, 7, 9, 10, 11, 12, 16, 17 and 18. Continuing self-development was categorised into four group domains that explained 63.4% of the cumulative contribution rate. The four domains were named “component 1: Q17 (iii), Q17 (vi), Q17 (iv), Q17 (v), Q17 (ii), Q17 (i), Q10 and Q16: undergraduate education and recognition”, “component 2: Q12 and Q11: self-improvement real behaviour of pharmacists”, “component 3: Q6: the ability to sense the needs of society” and “component 4: pharmacists’ behaviour as students”.

A principal component loading of 0.50 was used as a cut-off point, and the results of the analysis ranged from 0.501 to 0.842. The results of the factor analysis with principal component analysis are presented in Table 2.

## 4. Discussion

The role of Japanese pharmacists is changing with social needs. Japanese initial pharmacy education was revised based on social needs in 2022 and will be implemented in 2024. Increasing pressure on healthcare systems internationally has led to a radical change in pharmacy practice over the last decade, including an expansion not only of roles but also of scopes of practice, such as prescribing and assisting with the management of chronic conditions [21,22,23]. The notion of competence for this shift of roles is currently upheld mainly by continuing professional development (CPD) and revalidation models, whereby reflection is implicitly or explicitly utilised [24]. Our study suggests that education on competencies related to self-development must be provided in undergraduate education and postgraduate education in each university, especially regarding (a) the ability to identify one’s own problems and issues, (b) the ability to make plans to solve problems and issues, (c) the ability to carry out plans to solve problems and issues and (d) the ability to repeat the self-development steps.

In 2019, E. Mantzourani et al. examined the literature to uncover strategies employed to support reflection in healthcare environments and education with a particular focus on pharmacy and interrogated these strategies through the lens of self-assessment. As next steps for pharmacy education and practice, they found that reinforcing the notion of self-assessment, as argued by Eva and Regehr in 2005 [25], reflection-in-action and ongoing monitoring are essential for pharmacists to respond to the challenges of ever-changing roles and scopes of practice internationally [26]. Eva and Regehr also found that improving one’s self is intertwined with repeated, situation-specific relevant and reliable external assessment, such as feedback from experts. Therefore, it is clear that undergraduate pharmacy education needs to include education in self-reflection and self-development. In fact, regarding “Q16 Do you think you needed further education in your undergraduate education to continue self-development?” in our study, more than 50% of pharmacists answered “necessary”. However, self-development education is not sufficient in undergraduate education, and self-development education is used mainly in reflection papers. The results for self-reflection or self-assessment are fragmented and inconsistent, as these reflection papers are written for each subject. Therefore, comprehensive education on self-development or self-reflection is needed. In 2018, Matsushita K. et al. proposed the Pivotal Embedded Performance Assessment (PEPA) as a method for combining assessment at the course and program levels. The method limits the range of performance assessment to key courses directly linked to program goals and is placed at the critical juncture points of the curriculum while entrusting the assessment of other courses to the expert judgement of individual teachers [27,28]. Therefore, the introduction of PEPA at each milestone in pharmacy education allows the same assessment tool on self-reflection and self-development to be used in a 6-year program, and these assessments can be shared among teachers. Finally, if we can provide these assessments to pharmacy students, they will be able to see whether they have improved themselves.

Professional education requires self-development, and these competencies must be continued in pharmacists’ careers. Regarding the factors of self-development, our study revealed that self-development requires not only recognition but also the ability to act, behave correctly and perceive the needs of society. In particular, the factor of 4: pharmacists’ behaviour as students might be related to “in UCSF, disciplinary action taken against physicians by the Medical Board of California was associated with prior unprofessional behaviour when the physicians were students [14]” or “disciplinary action among practising physicians by medical boards was strongly associated with unprofessional behaviour in medical school [15]”. Moreover, regarding Q11, “How much time do you spend on continuing self-development related to pharmacist work during a week?”, the largest number of respondents answered “1–3 h”, which is by no means sufficient time for self-learning. Naturally, if they do not set goals, they will not know how many hours you need to study to reach their goals [29,30]. Then, regarding Q15, “Tools for continuing self-development”, respondents answered “at home using books purchased by themselves and web searches”, “study meeting of medical associations such as the Japanese Society of Hospital Pharmacists or Japan Pharmaceutical Association” and pharmaceutical companies’ (wholesalers) study meetings. Therefore, various places, including pharmacy universities, must provide learning tools or programs to develop pharmacists’ or students’ competencies. In addition, each university will be required to establish a system to monitor the lifelong learning status of graduates in order to assure the quality of pharmacists (including goal setting).

Our study found that many Japanese pharmacists recognise the need for self-development and have an attitude towards self-learning. On the other hand, the quality of pharmacists in Japan is not sufficiently ensured, as the setting of self-learning attainment levels and achievement is not clear and the timing of study is entirely up to the individual. Therefore, based on these data, it was considered necessary to further discuss lifelong education in Japan in the future.

The limitations of our study are that we conducted only one case study of Japanese pharmacists. However, as E. Mantzourani et al. stated in 2019, few studies have focused on reflective practices for pharmacy professionals. Therefore, we believe that the results of our study are useful to other countries’ self-development education.

## 5. Conclusions

CPD is essential for healthcare practitioners including pharmacists to stay current and up-to-date with the speed of new information becoming available every day, and in today’s environment, artificial intelligence and technology. During the spread of COVID-19, the role of pharmacists in the community has gradually changed [10]. In a society where swift responses and changes are needed, individuals who work as medical personnel need the ability to respond while remaining aware of the needs of society. To this end, we must continue to develop competencies for self-reflection and self-development among students and graduates through lifelong education. Universities have a role to play in ensuring the quality of the profession after the students they have trained themselves graduate and become pharmacists, and each healthcare organisation needs to work with universities to establish a system to ensure the quality of pharmacists. It was considered necessary for the national government to manage this system collectively and guarantee the quality of Japanese pharmacists that society will need in the future.

## Figures and Tables

**Table 1:** Characteristics of respondents (*n* = 529).

Questionnaire		*n*	%
Q1. Length of time as a pharmacist	1–5 years	7	1.3
	6−10 years	53	10.0
	11−15 years	93	17.6
	More than 16 years	376	71.1
Q2. Workplace	Hospital	208	39.3
	Community pharmacy	321	60.7
Q3. Undergraduate education coursework	4-Year initial pharmacy education	485	91.7
	6-Year initial pharmacy education (since 2006)	44	8.3
Q4. Do you have a qualification as a specialist pharmacist, certified pharmacist or doctorate (PhD)? (Multiple answers allowed)	Currently studying to become a specialist or certified pharmacist	57	10.8
	Special pharmacists	57	10.8
	Certified pharmacists	361	68.2
	PhD	31	5.9
	Nothing	104	19.7
Q5. Are you aware that the model core curriculum for pharmacy education for undergraduate students (6-year pharmacy education) will be revised in 2024?	No	334	63.1
	Yes	195	36.9
Q6. Do you feel that society’s needs for pharmacists are changing?	Not at all	5	0.9
	Not much	16	3.0
	Not very much	84	15.9
	A little	160	30.2
	Somewhat	184	34.8
	Quite a lot	80	15.1
Q7. As a pharmacist, do you feel the need to streamline your work with objects and enhance your work with people?	Not at all	5	0.9
	Not much	4	0.8
	Not very much	27	5.1
	A little	137	25.9
	Somewhat	244	46.1
	Quite a lot	112	21.2
Q9. When you were a student, did you study independently on a daily basis in addition to your assignments?	Not at all	0	0
	Not much	109	20.6
	Not very much	158	29.9
	A little	91	17.2
	Somewhat	42	7.9
	Quite a lot	129	24.4
Q10. Do you think continuing self-development is necessary as a pharmacist?	Not at all necessary	0	0
	Not necessary	2	0.4
	Not very necessary	9	1.7
	Slightly necessary	61	11.5
	Necessary	282	53.3
	Quite necessary	175	33.1
Q11. How much time do you spend on continuing self-development related to pharmacist work during a week?	0	49	9.3
	1−3 h	350	66.2
	4−6 h	76	14.4
	7−9 h	27	5.1
	More than 10 h	27	5.1
Q12. Are you constantly developing yourself (lack of knowledge and skills) as a pharmacist?	Not at all	15	2.8
	Not much	27	5.1
	Not very much	91	17.2
	A little	239	45.2
	Somewhat	126	23.8
	Quite a lot	31	5.9

**Table 2:** Principal component analysis (*n* = 439).

Question Items	Component 1	Component 2	Component 3	Component 4
Q17. What other skills do you think were needed in your undergraduate education? (iii) The ability to make plans to solve problems and issues	0.842	−0.272	0.043	−0.012
Q17. What other skills do you think were needed in your undergraduate education? (vi) The ability to repeat steps (i)–(v)	0.831	−0.285	−0.039	−0.022
Q17. What other skills do you think were needed in your undergraduate education? (iv) The ability to carry out plans to solve problems and issues	0.832	−0.293	0.029	−0.016
Q17. What other skills do you think were needed in your undergraduate education? (v) The ability to evaluate the consequences of actions	0.800	−0.275	−0.021	−0.019
Q17. What other skills do you think were needed in your undergraduate education? (ii) The ability to identify one’s own problems and issues	0.796	−0.274	0.071	0.099
Q17. What other skills do you think were needed in your undergraduate education? (i) The ability to reflect on oneself	0.726	−0.190	0.104	0.135
Q10. Do you think continuing self-development is necessary as a pharmacist?	0.537	0.472	−0.144	0.011
Q16. Do you think you needed further education in your undergraduate education to continue self-development?	0.501	0.082	−0.047	0.239
Q12. Do you constantly improve yourself as a pharmacist?	0.360	0.661	0.341	0.024
Q11. How much time do you spend on continuing self-development related to pharmacist work during a week?	0.330	0.586	0.403	0.139
Q6. Do you feel that society’s needs for pharmacists are changing?	0.423	0.382	−0.559	−0.256
Q9. When you were a student, did you study independently on a daily basis in addition to your assignments?	0.108	0.298	−0.320	0.752
Component variance	5.278	1.998	1.228	1.004
Contribution ratio	35.19	13.32	8.19	6.70
Cumulative contribution ratio	35.19	48.51	56.70	63.39

Communality was set to 1, and a principal component loading of 0.50 was used as the cut-off point.

## Data Availability

The data can be found in this paper.

## Supplementary Materials

The following supporting information can be downloaded at: https://www.mdpi.com/article/10.3390/pharmacy11020073/s1, Figure S1: Changes in the number of pharmacists (1994–2020).
