# Supplementary figures and images for: Japanese Pharmacists’ Perceptions of Self-Development Skills and Continuing Professional Development
Source: Pharmacy (Basel). 2023 Apr 11;11(2):73. doi: 10.3390/pharmacy11020073 (PMC10143704; doi:10.3390/pharmacy11020073)

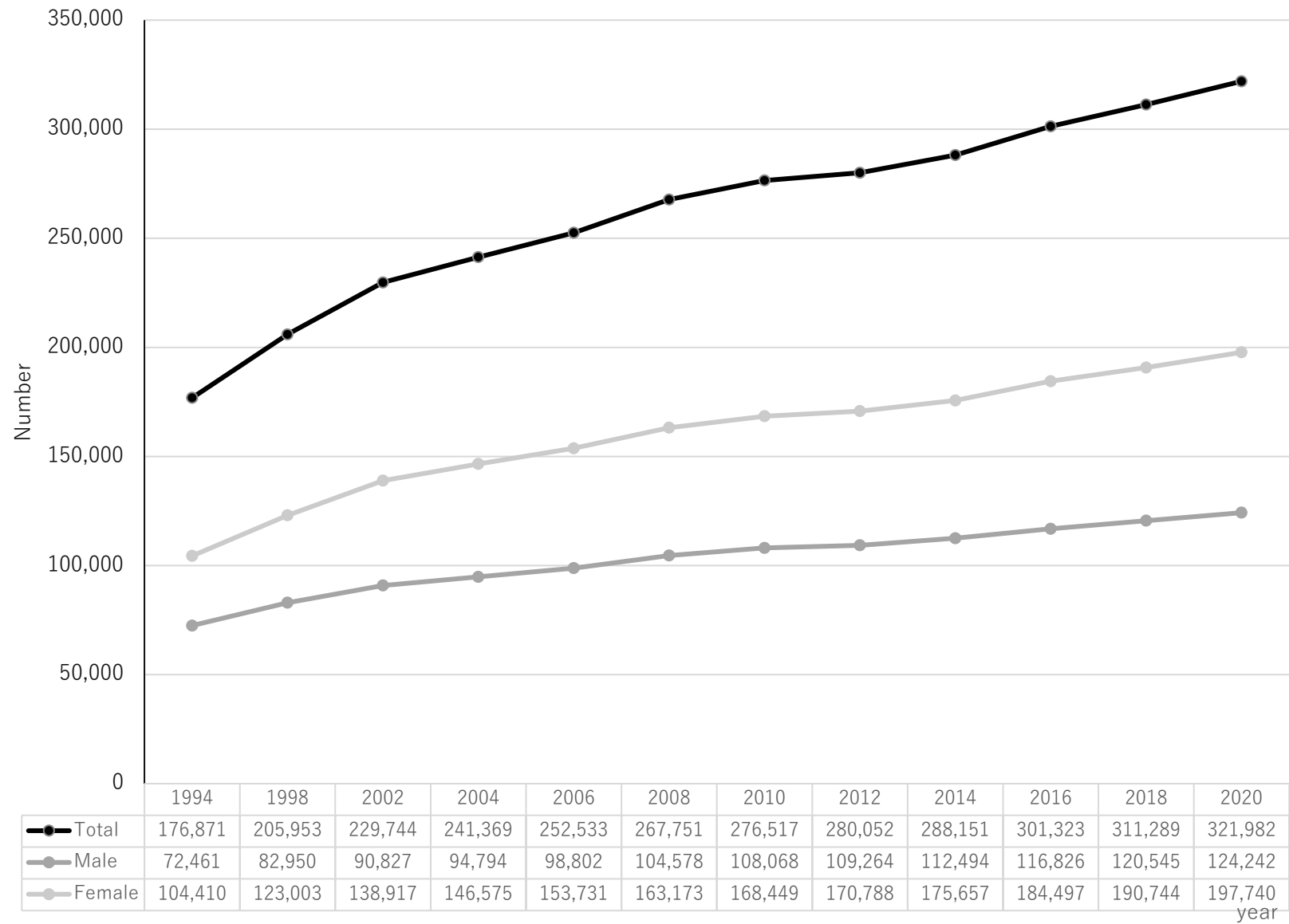

Supplementary Figure S1: Changes in the number of pharmacists (1994-2020)

Supplement: Supplementary file 1 [file pharmacy-11-00073-s001.zip › pharmacy-2326292-supplementary.pdf]
